# Transforming a Stem into a Bush

**DOI:** 10.1371/journal.pbio.1001476

**Published:** 2013-01-29

**Authors:** Amy Coombs

**Affiliations:** Freelance Science Writer, Chicago, Illinois, United States of America

**Figure:**
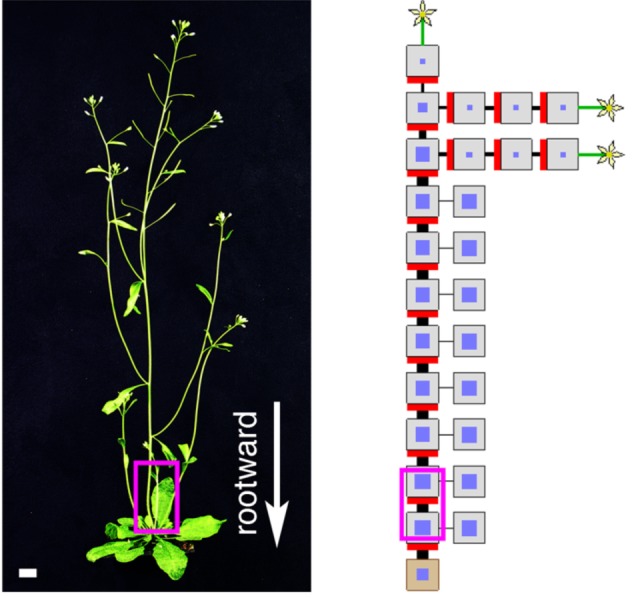
Computer-generated plants reveal how a self-organizing plant hormone network can balance growth across the shoot system.

Without careful pruning, a rose bush would grow into a single stem with leaves. This is why gardeners invest in a good pair of clippers—cutting the leading shoot helps activate axillary buds which typically form at the base of each leaf near the stem and give rise to new shoots.

Yet, axillary buds sometimes remain dormant despite pruning, and many plant species activate branching without a snip from the gardener. As to how a plant regulates this unpredictable branch growth, there are two competing theories that point to strigolactone, a recently discovered plant hormone. One theory suggests strigolactone primarily acts to regulate gene expression in axillary buds. New findings published this week in *PLOS Biology* support an alternative theory, which suggests strigolactone regulates bud growth by modulating the cellular flow of auxin, a plant growth hormone that influences development.

The findings explain how plants coordinate growth and development. According to a debated theory proposed by Ottoline Leyser and her colleagues at the University of Cambridge, the auxin transport network helps distribute branch growth across the plant in response to changing environmental conditions and resource availability.

This auxin transport canalization model suggests that cells in the axillary bud serve as a source of auxin while the stem and roots provide a sink. As auxin flows across the cell membrane, more exporter proteins are inserted into the plasma membrane. This increases the flow of auxin out of the cell, and leads to the insertion of even more exporter proteins. As this stream of auxin flows to the stem and roots, the axillary buds can potentially produce even more hormone.

Leyser believes this positive feedback loop triggers buds to grow into new shoots. After an axillary bud is activated, other buds must compete for access to sinks in the stem. The first few shoots dominate the local flow of auxin, making it harder for neighboring buds to establish the positive feedback of auxin export needed for activity. As a result, an axillary bud might not be able to activate after shoots start to grow elsewhere on the stem.

In previous work, Leyser and her colleagues showed that strigolactone reduces cellular levels of PIN1, a protein that exports auxin out of plant cells. As PIN1 accumulation requires a gene expressed in vascular associated cells, it's possible that strigolactone systemically inhibits canalization throughout the plant. But this theory conflicts with other research showing that strigolactone upregulates a transcription factor that inhibits bud growth, suggesting that strigolactone acts locally within the bud.

To test these alternative hypotheses, Leyser and her team grew mutant *Arabidopsis* plants incapable of synthesizing strigolactone or responding to its signal. By genetically modifying plants to produce fluorescently labeled PIN1 proteins, the researchers could track this auxin exporter in plant tissue. In keeping with prior findings, these strigolactone mutants grew more shoots and accumulated more PIN1. Similarly, treating mutants with synthetic strigolactone (GR24) reduced branching.

Yet, the story is a little more complex—it turns out strigolactone can both activate and repress shoot branching. Mutants weakly capable of transporting auxin showed elevated branching after a low dose of strigalactone, yet after a high dose, branching was reduced. In double mutants that lacked the ability to produce strigolactone and also suffered weak auxin transport, low doses of strigalactone reduced branching while high doses restored branching to levels of untreated plants. If strigalactone acts as a direct inhibitor of bud growth, these findings would be difficult to explain. It appears strigalactone can either promote or inhibit bud growth depending on the auxin transport status of the treated plant, although the underlying mechanisms remain unclear.

Leyser and her team further investigated the hormone's systemic function with a computational model, which suggested that branching can occur in one of two ways. First, if PIN1 proteins remain in the membrane after insertion, auxin transport can be rapidly established and new buds activate. In contrast, if the rate of PIN1 insertion is low, relatively less auxin flows out of the cell. This reduces competition for auxin transport down the stem, and an active bud is less likely to inhibit its neighbors. If strigolactone acts to promote removal of PIN1 proteins from the membrane, then the modeling results and experimental data together explain how strigolactone can either activate or repress shoot branching depending on the auxin transport status of the plants.

It's a new way of thinking about shoot growth, and it's not without controversy. Auxin canalization theory is contentious for at least two reasons—it assumes that auxin flux triggers PIN1 insertion and proposes that buds must export auxin in order to activate. It's not clear how a plant cell membrane can measure this flux.

While Leyser's findings don't resolve this question, they demonstrate a systemic role for strigalactone that supports the contentious auxin canalization model. The cellular flux of auxin may be influenced by the availability of sinks in the stem, and strigalactone may help regulate the abundance of PIN1 proteins needed to facilitate the flow of hormone in both locations. The picture that emerges is of the plant as a self-organizing system that balances its growth according to its environment. And as a result, a bush can grow from a stem.

**Shinohara N, Taylor C, Leyser O (2013) Strigolactone Can Promote or Inhibit Shoot Branching by Triggering Rapid Depletion of the Auxin Efflux Protein, PIN1, from the Plasma Membrane. doi:10.1371/journal.pbio.1001474**

